# The effects of the dialysis on the white matter tracts in patients with end-stage renal disease using differential tractography study

**DOI:** 10.1038/s41598-023-47533-7

**Published:** 2023-11-16

**Authors:** Bong Soo Park, Byeongo Choi, Chang Min Heo, Yoo Jin Lee, Sihyung Park, Yang Wook Kim, Junghae Ko, Dong Ah Lee, Kang Min Park

**Affiliations:** 1 Department of Internal Medicine, Haeundae Paik Hospital, Inje University College of Medicine, Busan, South Korea; 2 Department of Neurology, Haeundae Paik Hospital, Inje University College of Medicine, Haeundae-ro 875, Haeundae-gu, Busan, 48108 South Korea

**Keywords:** Neuroscience, Neurology

## Abstract

This study aimed to determine whether white matter tracts correlate with kidney function using correlation tractography, and to investigate the effects of dialysis on white matter tracts in patients with end-stage renal disease (ESRD) using differential tractography. Ten patients with ESRD, who had a glomerular filtration rate of < 15 mL/min/1.73 m^2^, were enrolled in this prospective study. Diffusion tensor imaging (DTI) was performed both before and after dialysis. We discovered that white matter tracts correlated with the estimated glomerular filtration rate based on pre- and post-dialysis DTI using correlation tractography and investigated the differences in the white matter tracts between pre- and post-dialysis DTI in patients with ESRD using differential tractography. Correlation tractography revealed no quantitative anisotropy of the white matter tracts that correlated with the estimated glomerular filtration rate in pre- and post-dialysis patients with ESRD. Differential tractography revealed significant differences in several white matter tracts, particularly the cingulum, thalamic radiation, corpus callosum, and superior longitudinal fasciculus, between pre- and post-dialysis DTI, which revealed increased diffusion density after dialysis. We demonstrated the significant effects of dialysis on several white matter tracts in patients with ESRD using differential tractography, which showed increased diffusion density after dialysis. In this study, we confirmed the effects of dialysis on brain structure, especially white matter tracts.

## Introduction

Chronic kidney disease (CKD) is defined as structural or functional kidney abnormalities lasting longer than three months^1^. It is a very common disease, with a global prevalence of 9.1 percent in the adult population^2^. End-stage kidney disease (ESRD) is characterized by a permanent loss of kidney function, usually necessitating long-term dialysis or kidney transplantation for survival. Patients with ESRD have a glomerular filtration rate^3^ below 15 mL/min per 1.73 m^2^.

Recent studies have actively explored brain changes in patients with ESRD^4–8^. One study, using voxel-based morphometry, found that patients with ESRD have predominantly diminished gray matter volumes, which are associated with cognitive dysfunction^9^. In addition, another study, using resting state functional magnetic resonance imaging (MRI) and graph theoretical analysis, demonstrated that patients with ESRD exhibited decreased global functional connectivity and alterations in brain network hubs when compared to both healthy and disease controls^8^. Diffusion tensor imaging (DTI) can reveal the microstructure of white matter, and reduced white matter integrity has been linked to patients with ESRD, as demonstrated by a DTI study^10^. Another DTI study also indicated that patients with ESRD have widespread white matter impairment^11^. However, despite several previous studies revealing abnormalities in white matter tracts in patients with ESRD undergoing dialysis, there have been few studies comparing white matter changes between pre- and post-dialysis in patients with ESRD^12^. This comparison can reveal what changes occur in the white matter after starting dialysis in patients with ESRD, and which white matter tract has the most influence. In addition, the presence or absence of brain function recovery after hemodialysis can be determined.

Correlation and differential tractography have recently been introduced as new tractography modalities for studying white matter tracts. Specifically, differential tractography employs repeated DTI scans of the same subject at various time points to map precise segments of fiber pathways with neuronal injury in longitudinal studies. As a quantitative and objective method, it has metrics for monitoring neuronal injury in a single subject without considering inter-subject and group variability, allowing for individual-level diagnostic and prognostic evaluation of brain diseases^13–15^. Differential tractography, in contrast to conventional tractography, which maps all existing pathways, can identify specific pathway segments exhibiting longitudinal changes^13,15–17^. Differential tractography integrates the “tracking-the-difference” paradigm as opposed to the “tracking-the-existence” used in the conventional setting. Differential tractography greatly increases the sensitivity and specificity of diffusion metrics by aggregating results along white matter pathways^13–15^. However, no studies have applied correlation and differential tractography in patients with CKD.

This study aimed to determine whether white matter tracts correlate with kidney function using correlation tractography and to investigate the effects of dialysis on white matter tracts in patients with ESRD using differential tractography. We initially hypothesized that some white matter tracts were associated with kidney function and that their diffusion density could change after dialysis.

## Results

### Demographic and clinical characteristics in patients with end-stage renal disease

Table 1 shows the demographic and clinical characteristics of patients with ESRD. Of the 10 patients with ESRD, half of the patients were male. Fifty percent of the patients had diabetes and 90% had hypertension.

**Table 1:** Demographic and clinical characteristics in patients with end-stage renal disease.

Clinical data	Patients with end-stage renal disease (N = 10)
Demographic data	
Age, years (SD)	57.4 (12.0)
Male, N (%)	5 (50.0)
Comorbidities	
Diabetes mellitus, N (%)	5 (50.0)
Hypertension, N (%)	9 (90.0)
Laboratory data	
Hemoglobin, g/dL (SD)	9.0 (1.6)
Hematocrit, % (SD)	28.2 (5.0)
Estimated glomerular filtration rate, mL/min/1.73 m^2^ (SD)	4.1 (2.2)
Protein, g/dL (SD)	5.8 (0.3)
Albumin, g/dL (SD)	3.3 (0.3)
BUN, mg/dL (SD)	96.7 (33.6)
Creatinine, mg/dL (SD)	11.7 (4.6)
Sodium, mmol/L (SD)	136.1 (3.1)
Potassium, mmol/L (SD)	4.6 (0.9)
Chloride, mmol/L (SD)	102.8 (6.2)
Calcium, mg/dL (SD)	7.2 (0.8)
Phosphate, mg/dL (SD)	6.9 (2.2)
Total CO_2_ contents, mmol/L (SD)	17.1 (4.3)

### Correlation tractography

Correlation tractography revealed that there were no QA of the white matter tracts that correlated with the estimated glomerular filtration rate in patients with ESRD pre- and post-dialysis after multiple corrections, suggesting that no white matter tracts were associated with kidney function in patients with ESRD.

### Differential tractography

**Figure 1:**
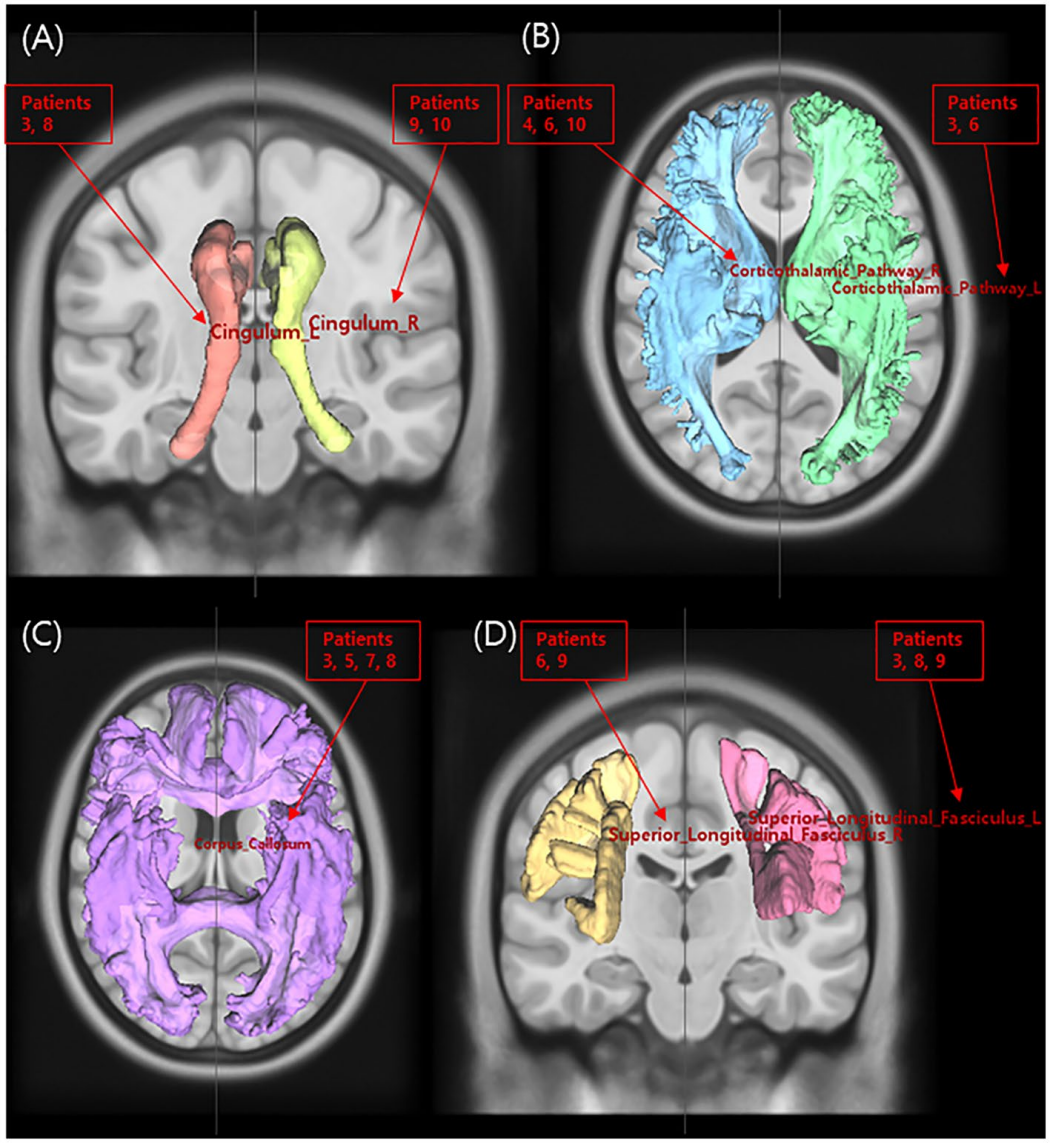
The results of the differential tractography. The most common white matter tracts with significant differences between diffusion tensor images pre- and post-dialysis are the cingulum (**A**), thalamic radiation (**B**), corpus callosum (**C**), and superior longitudinal fasciculus (**D**), which reveals an increased diffusion density after dialysis. The red numbers indicating a tract are the number of patients involved in that tract.

**Figure 2:**
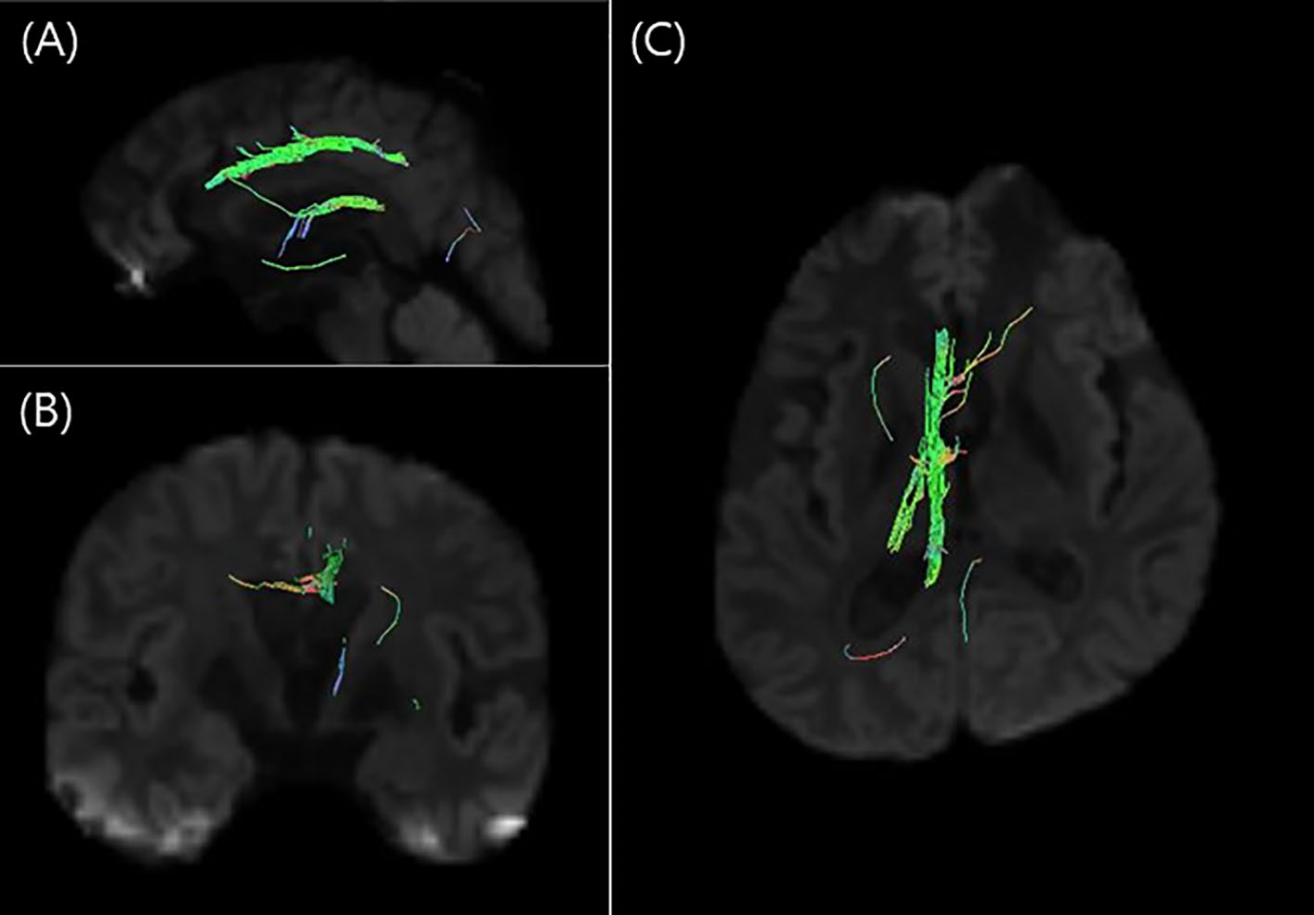
The example of the differential tractography results in a patient with end-stage renal disease (Patient 3). Differential tractography showed significant changes in the white matter tracts, including the left cingulum parolfactory, left cingulum frontoparietal, left superior longitudinal fasciculus, left thalamic radiation anterior, left thalamic radiation superior, left fornix, left cingulum frontoparippocampal, corpus callosum tapetum, and corpus callosum forceps minor, between pre- and post-dialysis (sagittal (**A**), coronal (**B**), and axial (**C**) views).

Differential tractography revealed that the most common white matter tracts with significant differences between DTI pre- and post-dialysis were the cingulum, thalamic radiation, corpus callosum, and superior longitudinal fasciculus, which revealed an increased diffusion density after dialysis (Fig. 1). The next most common changes were observed in the inferior fronto-occipital fasciculus, corticospinal tract, fornix, medial lemniscus, and the reticular tract. The corticostriatal tract, dentatorubrothalamic tract, inferior longitudinal fasciculus, and middle cerebellar peduncle are white matter tracts, and differences were observed only in each patient. Table 2 shows the results of differential tractography for each patient, and Fig. 2 shows the results of one example of differential tractography. However, no white matter tracts showed decreased diffusion density after dialysis.

**Table 2:** The results of the differential tractography between end-stage renal disease patients before and after dialysis.

Patients with ESRD	Altered white matter tracts
Patient 1	Lt. corticospinal tract, Lt. medial lemniscus, Rt. fornix, Rt. medial lemniscus, Lt. dentatorubrothalamic tract
Patient 2	Rt. corticostriatal tract posterior part
Patient 3	Lt. cingulum parolfactory part, Lt. cingulum fronto-parietal part, Lt. superior longitudinal fasciculus, Lt. thalamic radiation anterior part, Lt. thalamic radiation superior part, Lt. fornix, Lt. cingulum fronto-parahippocampal part, Corpus callosum tapetum, Corpus callosum forceps minor
Patient 4	Rt. inferior fronto-occipital fasciculus, Rt. thalamic radiation anterior part
Patient 5	Lt. inferior fronto-occipital fasciculus, Corpus callosum forceps minor
Patient 6	Lt. inferior fronto-occipital fasciculus, Rt. reticular tract, Rt. superior longitudinal fasciculus, Lt. thalamic radiation anterior part, Rt. thalamic radiation anterior part
Patient 7	Rt. reticular tract, Rt. fornix, Corpus callosum forceps major
Patient 8	Lt. superior longitudinal fasciculus, Lt. cingulum fronto-parietal part, Lt. inferior longitudinal fasciculus, Lt. cingulum fronto-parahippocampal part, Lt. inferior fronto-occipital fasciculus, Corpus callosum tapetum, Rt. corticostriatal tract superior part
Patient 9	Rt. superior longitudinal fasciculus, Rt. cingulum fronto-parietal part, Lt. superior longitudinal fasciculus, Rt. cingulum fronto-parahippocampal part
Patient 10	Rt. reticular tract, Rt. medial lemniscus, Rt. corticospinal tract, Rt. thalamic radiation posterior part, Middle cerebellar peduncle, Rt. cingulum parahippocampo-parietal part

*ESRD* end-stage renal disease, *Lt* left, *Rt* right.

We found no white matter tracks showing significant differences of the FA between pre- and post- dialysis in patients with ESRD using voxel-based analysis after multiple corrections (Supplementary Table 1).

## Discussion

The main finding of this study was that white matter tracts were not correlated with kidney function in patients with ESRD undergoing pre-dialysis. However, significantly different white matter tracts showed increased diffusion density after dialysis, particularly the cingulum, thalamic radiation, corpus callosum, and superior longitudinal fasciculus in patients with ESRD.

We could not find any correlation between white matter tracts and kidney function in ESRD patients on pre- and post-dialysis using correlation tractography. This result is consistent with those of previous studies involving 35 patients with ESRD. This DTI study revealed no correlation between fractional anisotropy (FA) values and serum creatinine, serum urea levels, duration of dialysis, or disease duration^11^. However, our study differs from previous studies, even with the same correlation analysis. In this study, we reconstructed the DTI data using the GQI method. Diffusion tensor reconstruction can yield several diffusivity measures, including FA, axial diffusivity (AD), radial diffusivity (RD), and mean diffusivity (MD)^18^. FA are some of the most important measurements derived from DTI data. It quantifies the degree of anisotropy or directionality of intracellular water diffusion. Anisotropy refers to the tendency of water molecules to diffuse more readily in certain directions, which indicates the organization and integrity of the microstructures^18,19^. On the other hand, the GQI reconstruction method can provide QA and an isotropic diffusion component derived based on diffusion density^20^. QA is computed using peak orientations on a spin distribution function, where each orientation defines a specific QA value. Notably, QA is defined per fiber orientation, whereas FA is defined per voxel. FA can decrease in the presence of crossing fibers, whereas QA is less affected. QA is also less sensitive to the partial volume effects of crossing fibers with better resolution^21,22^. Therefore, we used QA measures in our study because it is more meaningful than FA.

We also used QA in differential tractography, which revealed several white matter tracts with increased diffusion density after dialysis, particularly the cingulum, thalamic radiation, corpus callosum, and superior longitudinal fasciculus in patients with ESRD. Although this is the first study to demonstrate increased QA in some white matter tracts after hemodialysis, several similar previous studies have used FA values. In a previous prospective study, 11 patients with ESRD who were on the waiting list for a kidney transplant (KT) were followed post-transplant^23^. The authors evaluated the patients’ cognitive function using a battery of neuropsychological tests and evaluated their brain white matter integrity using DTI before and three months after KT. In the post-transplant period, cognitive function, particularly memory and executive function, showed improvements along with white matter integrity in the tracts associated with memory and executive function^23^. In another study, connectivity changes after kidney replacement therapy were investigated using resting state functional MRI and DTI. The study examined the patterns of large-scale complex networks in patients with ESRD after KT. At one month post-KT, both functional and structural networks exhibited increased node degree and node efficiency compared to pre-KT, with further increases at 6 months post-KT^24^. By combining these preceding studies with our present findings, it becomes evident that kidney replacement therapy can effectively restore many structural and functional parts of the brain, including white matter tracts, in comparison to pre-kidney replacement therapy.

There are several possible reasons for the increase in the QA value after dialysis^5,7,23,25,26^. The ESRD causes the accumulation of waste products and toxins in the bloodstream because the kidneys are unable to filter them efficiently. The removal of these harmful substances from blood through dialysis reduces their impact on brain function. In addition, by restoring fluid and electrolyte balance, dialysis assists in maintaining the body's fluid and electrolyte balance^26^. This is essential for brain health, as imbalances can lead to problems such as impairment of brain function. Furthermore, chronic inflammation can have detrimental effects on brain function and is associated with ESRD reduced by dialysis, which may contribute to improved cognitive outcomes.

Interestingly, we found that QA in the cingulum, thalamic radiation, corpus callosum, and superior longitudinal fasciculus was especially increased in the post-dialysis state compared to the pre-dialysis state. The cingulum connects various portions of the cingulate gyrus to other brain regions, thereby facilitating the exchange of information related to emotional and cognitive processes. The cingulate gyrus is an essential part of the limbic system that regulates emotions and motivation. It is also related to executive functions, which include higher-order cognitive processes such as problem solving, decision-making, and planning^27^. Therefore, an increase in the diffusion density of the cingulum after dialysis may be associated with an increase in the function of the limbic system and cognitive function. The corpus callosum facilitates communication and coordination between the hemispheres, enabling them to function as a unified entity. It enables the transfer of information between the left and right hemispheres, such as sensory inputs, motor commands, and cognitive processes^28^. Thalamic radiation refers to a network of white matter fibers that connect the thalamus to various regions of the cerebral cortex. The thalamus is a central structure situated deep within the brain that transmits sensory and motor signals between various brain regions^29^. The superior longitudinal fasciculus is a major white matter tract in the brain that connects the frontal, parietal, and occipital lobes of the cerebral cortex. It is one of several longitudinal fasciculi in the brain that serve as a crucial pathway for communication between distal brain regions^30^. Therefore, we can expect an overall increase in structural and functional connectivity after dialysis in patients with ESRD. Interestingly, a previous study demonstrated significant increases of FA after kidney transplantation, especially in the regions of the corpus callosum, forceps minor, and cingulate gyrus^23^. The white matter where QA was elevated after hemodialysis in this study and the areas where FA was elevated in the previous study were very consistent, which suggested that regardless of the type of renal replacement therapy, the effect on the white matter tract is similar. Furthermore, in this study, we found that only the unilateral white matter tract was changed in each patient after hemodialysis, rather than changing bilateral white matter tracts. Although it was different for each patient whether the change was right or left hemisphere, it was found that bilateral white matter tracts were not involved at the same time. However, the plausible reason for this finding was not clear. It is necessary to include and analyze large sample size of the patients with ESRD and analyze it in order to clarify this findings.

This study had some limitations. First, our sample size was small, with only 10 enrolled patients with ESRD. However, since this was a longitudinal follow-up study, enrolling a larger number of patients proved challenging because the first DTI was performed immediately before dialysis, and the second MRI was performed 3 months after dialysis in each patient. Despite the limited sample size, the results remained meaningful even after multiple corrections, indicating their significance. Further studies with large sample size are needed to confirm our findings. Second, we could not include a normal control group and thus performed two DTI scans. This limitation prevented us from directly comparing changes in the white matter tract over time in patients with ESRD undergoing dialysis with a healthy control group. Third, we did not administer cognitive function tests before or after dialysis. Therefore, we could not confirm whether cognitive function improved in correlation with the QA of the white matter tract after dialysis. After hemodialysis, it was expected that cognitive function would improve along with white matter change. Previous study showed that cognitive dysfunction in patients with ESRD could improve with dialysis because of partial correction of anemia, control of extracellular volume excess, and removal of uremic toxin^31^. Third, we compared DTI between before dialysis and three months after dialysis, but three months after dialysis may be a relatively short time to observe structural changes in the brain. After hemodialysis begins, the brain's metabolic function probably change more quickly, whereas structural changes could likely change more clearly over a longer period of time. Fourth, we could not extract all white matter tract data for each patient based on the differential tractography method using the DSI studio program. Lastly, another limitation of this study was the inability to control the effect of comorbidities such as diabetes or hypertension on DTI.

We demonstrated the significant effects of dialysis on several white matter tracts in patients with ESRD using differential tractography, which showed an increased diffusion density after dialysis. In this study, we confirmed the effects of dialysis on brain structure, especially white matter tracts.

## Methods

### Participants

This study was performed in accordance with the ethical standards laid down in the 1964 Declaration of Helsinki and its later amendments or comparable ethical standards. The study was approved by the institutional review board of the Haeundae Paik Hospital. Informed consent was obtained from all participating subjects. All methods were carried out in accordance with relevant guidelines and regulations. We prospectively enrolled 10 patients with ESRD who had a glomerular filtration rate^3^ of < 15 mL/min/1.73 m^2^. Patients who were in need of but had never undergone dialysis before. All patients had normal brain MRI without any structural lesions at the time of enrollment.

### Diffusion tensor imaging scan

The first DTI MRI was performed immediately before the start of dialysis (pre-dialysis) and the second DTI MRI was performed 3 months after the start of dialysis (post-dialysis). Therefore, two DTIs were performed for each patient. All DTIs were performed using the same MRI scanner and protocol. All DTI scans were performed using a 3.0 T MRI scanner (AchievaTx; Phillips Healthcare, Best, Netherlands) equipped with a 32-channel head coil. DTI was performed using spin-echo single-shot echo-planar pulse sequences with a total of 32 different diffusion directions (repetition time/echo time, 8620/85 ms; flip angle, 90°; slice thickness = 2.25 mm, acquisition matrix, 120 × 120; field of view, 240 × 240 mm^2^; and *b*-value, 1000 s/mm^2^). Three-dimensional T2- and T1-weighted imaging were also performed to examine the presence or absence of structural brain lesions.

### Correlation tractography and differential tractography

**Figure 3:**
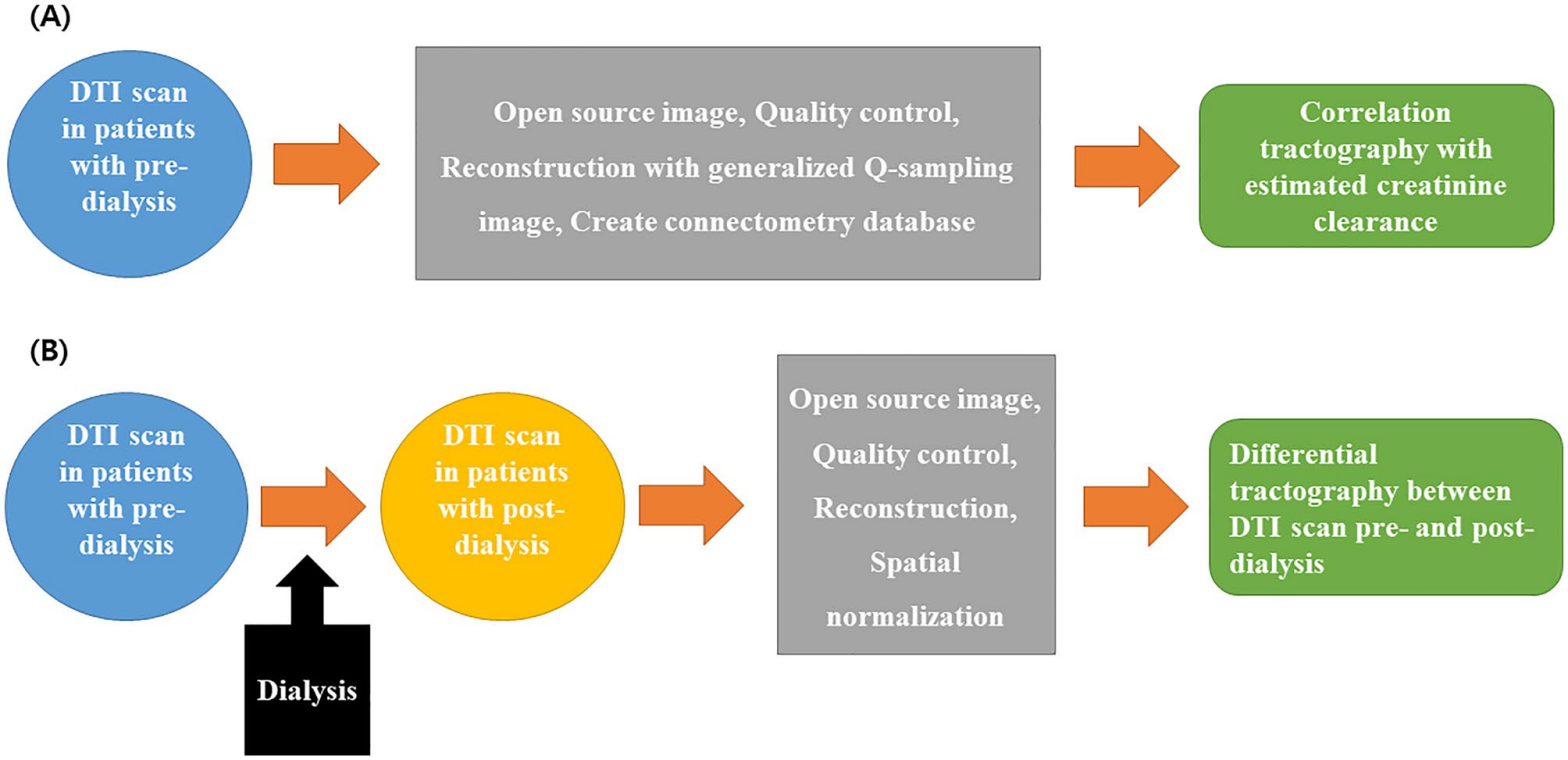
The process for the correlation tractography and differential tractography in this study. Diffusion tensor images in patients before dialysis were used to conduct correlation tractography (**A**), and diffusion tensor images in patients before and after dialysis were used to conduct differential tractography (**B**).

Figure 3 shows the DTI process used for correlation and differential tractography. DTI in pre- and post-dialysis patients was used to conduct correlation tractography, and DTI in pre- and post- dialysis patients was used to conduct differential tractography. We used the DSI studio tractography software (version 2023, http://dsi-studio.labsolver.org) in this study.

First, we preprocessed the DTI images, which included open-source images, corrected the eddy current and phase distortion artifacts, and set up a mask (thresholding, smoothing, and defragmentation). Then, the DTI data were reconstructed using the generalized q-sampling imaging (GQI) method with a diffusion sampling length ratio of 1.25^20^. We could obtain a measure of quantitative anisotropy (QA), which is a GQI-based metrics^32^. It was extracted as the local connectome fingerprint and used in the connectometry analysis. A non-parametric Spearman partial correlation was used to derive the correlation, and the effects of sex and age were removed using a multiple regression model. A T-score threshold of 2.5 was assigned and tracked using a deterministic fiber tracking algorithm to obtain correlational tractography^32^. A seeding region was placed in the whole brain. The tracks were filtered by topology-informed pruning with four iterations^33^. A false discovery rate threshold of 0.05 was used to select tracks to conduct multiple corrections. A total of 4000 randomized permutations were applied to the group labels to obtain a null distribution of the track length. We discovered that white matter tracts correlated with the estimated glomerular filtration rate based on pre- and post-dialysis DTI using correlation tractography.

We investigated the differences in white matter tracts between pre- and post-dialysis DTI using differential tractography in patients with ESRD. We used DTI data for pre-dialysis. After DTI reconstruction using the GQI method, a deterministic fiber tracking algorithm was used with augmented tracking strategies to improve reproducibility. Tracking index was QA. The angular threshold was randomly selected from 15° to 90°. The step size was randomly selected in 0.5 voxel of 1.5 voxels. Tracks shorter than 30 mm or longer than 300 mm were discarded. A total of 1,000,000 seeds were planted. Subsequently, we selected the post-dialysis DTI. We conducted differential tractography between pre- and post-dialysis DTI with a threshold of 0.2^13–15^.

Furthermore, we investigated the difference of the FA between pre- and post- dialysis in patients with ESRD using voxel-based analysis. After reconstruction of the DTI data, we conducted the normalization of the data to the ICBM 152 template. We obtained the FA values based on the HCP 842 tractography, derived from data acquired by the Human Connectome Project, using voxel-based analysis. We analzyed the difference of the FA between pre- and post- dialysis using Wilcoxon test, and applied a false discovery rate for multiple corrections.

### Supplementary Information

Supplementary Table 1.

## Data Availability

The datasets generated during and/or analyzed during the current study are available from the corresponding author on reasonable request.
