# Supplementary material for: The effects of the dialysis on the white matter tracts in patients with end-stage renal disease using differential tractography study
Source: Sci Rep. 2023 Nov 16;13:20064. doi: 10.1038/s41598-023-47533-7 (PMC10654401; doi:10.1038/s41598-023-47533-7)
Supplement: Supplementary file 1 — Supplementary Table 1. [file 41598_2023_47533_MOESM1_ESM.docx]

**Supplementary Table 1.** Differences of the fractional anisotropy between pre- and post- dialysis in patients with end-stage renal disease using voxel-based analysis

|  | Pre-dialysis | Post-dialysis |  |
| --- | --- | --- | --- |
| Tract | median fractional anisotropy | median fractional anisotropy | *p*-value |
| Lt. Acoustic Radiation | 0.454 | 0.452 | 0.106 |
| Rt. Acoustic Radiation | 0.457 | 0.449 | 0.014 |
| Anterior Commissure | 0.357 | 0.353 | 0.922 |
| Lt. Arcuate Fasciculus | 0.327 | 0.323 | 0.322 |
| Rt. Arcuate Fasciculus | 0.315 | 0.318 | 0.846 |
| Lt. Central Tegmental Tract | 0.478 | 0.481 | 0.375 |
| Rt. Central Tegmental Tract | 0.502 | 0.520 | 0.432 |
| Lt. Cerebellum | 0.219 | 0.221 | 0.322 |
| Rt. Cerebellum | 0.205 | 0.205 | 0.770 |
| Lt. Cingulum | 0.246 | 0.254 | 0.770 |
| Rt. Cingulum | 0.307 | 0.305 | 1.000 |
| Corpus Callosum | 0.320 | 0.319 | 0.492 |
| Lt. Cortico Spinal Tract | 0.424 | 0.415 | 0.027 |
| Rt. Cortico Spinal Tract | 0.407 | 0.403 | 0.492 |
| Lt. Cortico Striatal Pathway | 0.330 | 0.325 | 0.432 |
| Rt. Cortico Striatal Pathway | 0.327 | 0.323 | 0.492 |
| Lt. Corticothalamic Pathway | 0.336 | 0.335 | 0.322 |
| Rt. Corticothalamic Pathway | 0.352 | 0.348 | 0.492 |
| Lt. Dorsal Longitudinal Fasciculus | 0.326 | 0.347 | 0.695 |
| Rt. Dorsal Longitudinal Fasciculus | 0.386 | 0.358 | 0.492 |
| Lt. Extreme Capsule | 0.374 | 0.371 | 0.193 |
| Rt. Extreme Capsule | 0.312 | 0.321 | 0.625 |
| Lt. Fornix | 0.250 | 0.256 | 1.000 |
| Rt. Fornix | 0.211 | 0.211 | 0.695 |
| Lt. Frontal Aslant Tract | 0.326 | 0.318 | 0.193 |
| Rt. Frontal Aslant Tract | 0.277 | 0.279 | 0.492 |
| Lt. Frontopontine Tract | 0.380 | 0.372 | 0.193 |
| Rt. Frontopontine Tract | 0.318 | 0.316 | 0.193 |
| Lt. Inferior Cerebellar Peduncle | 0.367 | 0.367 | 1.000 |
| Rt. Inferior Cerebellar Peduncle | 0.368 | 0.371 | 0.770 |
| Lt. Inferior Fronto Occipital Fasciculus | 0.322 | 0.328 | 0.922 |
| Rt. Inferior Fronto Occipital Fasciculus | 0.321 | 0.324 | 0.770 |
| Lt. Inferior Longitudinal Fasciculus | 0.331 | 0.329 | 1.000 |
| Rt. Inferior Longitudinal Fasciculus | 0.353 | 0.340 | 0.375 |
| Lt. Lateral Lemniscus | 0.302 | 0.296 | 0.695 |
| Rt. Lateral Lemniscus | 0.276 | 0.267 | 0.695 |
| Lt. Medial Lemniscus | 0.495 | 0.497 | 0.557 |
| Rt. Medial Lemniscus | 0.470 | 0.473 | 0.275 |
| Lt. Medial Longitudinal Fasciculus | 0.478 | 0.471 | 0.275 |
| Rt. Medial Longitudinal Fasciculus | 0.478 | 0.441 | 0.432 |
| Middle Cerebellar Peduncle | 0.414 | 0.412 | 0.557 |
| Lt. Middle Longitudinal Fasciculus | 0.282 | 0.283 | 0.492 |
| Rt. Middle Longitudinal Fasciculus | 0.335 | 0.334 | 0.922 |
| Lt. Occipitopontine Tract | 0.428 | 0.432 | 0.432 |
| Rt. Occipitopontine Tract | 0.427 | 0.419 | 0.770 |
| Lt. Optic Radiation | 0.351 | 0.348 | 0.770 |
| Rt. Optic Radiation | 0.384 | 0.386 | 0.322 |
| Lt. Parietopontine Tract | 0.431 | 0.430 | 0.131 |
| Rt. Parietopontine Tract | 0.397 | 0.396 | 0.193 |
| Posterior Commissure | 0.300 | 0.281 | 0.846 |
| Lt. Rubrospinal Tract | 0.467 | 0.454 | 0.695 |
| Rt. Rubrospinal Tract | 0.439 | 0.422 | 0.770 |
| Lt. Spinothalamic Tract | 0.456 | 0.444 | 0.492 |
| Rt. Spinothalamic Tract | 0.433 | 0.434 | 0.846 |
| Superior Cerebellar Peduncle | 0.442 | 0.439 | 0.557 |
| Lt. Superior Longitudinal Fasciculus | 0.302 | 0.300 | 0.375 |
| Rt. Superior Longitudinal Fasciculus | 0.298 | 0.298 | 0.492 |
| Lt. Temporopontine Tract | 0.461 | 0.445 | 0.557 |
| Rt. Temporopontine Tract | 0.409 | 0.409 | 0.275 |
| Lt. U Fiber | 0.275 | 0.277 | 0.375 |
| Rt. U Fiber | 0.259 | 0.258 | 0.375 |
| Lt. Uncinate Fasciculus | 0.264 | 0.271 | 0.432 |
| Rt. Uncinate Fasciculus | 0.295 | 0.295 | 1.000 |
| Lt. Vertical Occipital Fasciculus | 0.318 | 0.308 | 0.065 |
| Rt. Vertical Occipital Fasciculus | 0.309 | 0.303 | 0.492 |
